# A Selective PPARγ Modulator Reduces Hepatic Fibrosis

**DOI:** 10.3390/biology9070151

**Published:** 2020-07-02

**Authors:** Benita L. McVicker, Frederick G. Hamel, Ronda L. Simpson, Robert G. Bennett

**Affiliations:** 1 Research Service, VA Nebraska-Western Iowa Health Care System, Omaha, NE 68105, USA; bmcvicker@unmc.edu (B.L.M.); Frederick.Hamel@va.gov (F.G.H.); rlwhite@unmc.edu (R.L.S.); 2 Department of Internal Medicine, University of Nebraska Medical Center, Omaha, NE 68198, USA; 3 Department of Pharmacology and Experimental Neuroscience, University of Nebraska Medical Center, Omaha, NE 68198, USA; 4 Department of Biochemistry and Molecular Biology, University of Nebraska Medical Center, Omaha, NE 68198, USA

**Keywords:** hepatic fibrosis, peroxisome proliferator-activated receptor gamma, cirrhosis

## Abstract

Hepatic fibrosis is the accumulation of excess collagen as a result of chronic liver injury. If left unabated, hepatic fibrosis can lead to the disruption of the liver architecture, portal hypertension, and increased risk of progression to cirrhosis and hepatocellular carcinoma. The thiazolidinedione class of antidiabetic drugs, through their target peroxisome proliferator-activated receptor γ (PPARγ), have protective effects against liver fibrosis, and can inhibit the profibrotic activity of hepatic stellate cells, the major collagen-producing liver cells. However, these drugs have been ineffective in the treatment of established fibrosis, possibly due to side effects such as increased weight and adiposity. Recently, selective PPARγ modulators that lack these side effects have been identified, but their role in treating fibrosis has not been studied. In this study, we tested the effectiveness of one of these selective modulators, SR1664, in the mouse carbon tetrachloride model of established hepatic fibrosis. Treatment with SR1664 reduced the total and type 1 collagen content without increasing body weight. The abundance of activated hepatic stellate cells was also significantly decreased. Finally, SR1664 inhibited the profibrotic phenotype of hepatic stellate cells. In summary, a selective PPARγ modulator was effective in the reduction of established hepatic fibrosis and the activated phenotype of hepatic stellate cells. This may represent a new treatment approach for hepatic fibrosis.

## 1. Introduction

Hepatic fibrosis results from chronic liver injury due to a variety of causes, including hepatitis, alcohol overuse, and non-alcoholic fatty liver disease [1]. A key feature of hepatic fibrosis is the accumulation of excess fibrillar collagen, resulting from increased production of collagens and decreased extracellular matrix degradation. If the cause of the injury is not removed, the fibrosis will worsen, with disruption of the normal liver architecture, portal hypertension, organ dysfunction, and increased risk of progression to cirrhosis and hepatocellular carcinoma. To date, no effective treatments have been developed for hepatic fibrosis.

The major cells responsible for collagen production after liver injury are the hepatic stellate cells (HSCs), non-parenchymal cells that reside in the perisinusoidal region [2]. These cells are normally quiescent, retinoid-storing cells. However, once activated, they express the myofibroblastic marker smooth muscle α-actin (SMA), and produce increased amounts of fibrillar collagen and tissue inhibitors of metalloproteinases (TIMPs), tipping the balance toward the accumulation of collagen [3]. Due to these properties, these cells are a prime target for potential hepatic fibrosis treatments [4]. Numerous agents have been identified as possible HSC-targeting drugs, including agonists of peroxisome proliferator-activated receptor γ (PPARγ) [5]. The antidiabetic thiazolidinedione drugs (e.g., rosiglitazone and pioglitazone) are potent activators of PPARγ that, in addition to their insulin-sensitizing qualities, can protect against the development of fibrosis in prevention models [6,7,8,9,10,11]. Part of their mechanism of action in this latter respect is the inhibition of the activated phenotype of HSCs and the promotion of a more quiescent state [6,12,13,14]. Despite these promising findings, the thiazolidinedione drugs were ineffective in treating established fibrosis [15,16]. This class of drug also has undesirable side effects, such as edema, weight gain due to increased adipose tissue mass, and bone loss. Therefore, their use has largely been limited to increase insulin sensitivity in diabetes and fatty liver disease.

More recent studies have identified other PPARγ-binding agents that retain the insulin-sensitizing effects, but do not trigger the strong adipogenic program associated with the unwanted side effects of the thiazolidinediones. These agents, which include partial agonists or non-agonists, serve to block the inhibitory phosphorylation of PPARγ by cyclin-dependent protein kinase 5 (CDK5), resulting in enhanced basal activity of PPARγ associated with promoting insulin-sensitizing genes [17]. One of these drugs, SR1664, was found to effectively improve insulin sensitivity in a mouse model, without inducing weight gain or bone loss [18]. To date, there have been no studies to determine if selective modulation results in the antifibrotic effects associated with full agonism of PPARγ. In this study, we used a mouse model of established hepatic fibrosis to determine if SR1664 treatment resulted in reduced disease, and an HSC cell line to determine if SR1664 had direct effects on HSCs.

## 2. Materials and Methods

Animals: All animal experiments were conducted in accordance with the Guide for the Care and Use of Laboratory Animals, and were approved by the VA Nebraska-Western Iowa Health Care System’s Institutional Animal Care and Use Committee. Male C57BL/6J mice (Jackson Laboratories, Bar Harbor, ME, USA) aged 8 weeks were maintained with free access to food and water for the duration of the study. The mice were randomly assigned to treatment groups (*n* = 6 per group). To induce hepatic fibrosis, carbon tetrachloride (CCl_4_) diluted 1:4 in sunflower oil was injected intraperitoneally at a dose of 1 mL/kg body weight twice a week for 6 weeks, as described previously [19]. The control mice were injected with oil alone. For the final 2 weeks, the mice received daily oral administration of SR1664 (Cayman Chemical, Ann Arbor, MI, USA) mixed with peanut butter to deliver a dose of 20 mg/kg body weight, which was previously shown to be an optimal dose to reduce insulin resistance in mice [18]. The control mice received peanut butter alone. After 6 weeks, 72 h after the last CCl_4_ injection, the mice were humanely euthanized for blood and tissue collection.

Serum and tissue determinations: Serum alanine transaminase (ALT) and aspartate transaminase (AST) were measured by the VA Nebraska-Western Iowa Health Care System’s Clinical Chemistry Service using standard assays. The serum total adiponectin level was determined using an ELISA kit (Alpco, Salem, NH, USA) according to the manufacturer’s instructions. Liver hydroxyproline content was determined as described previously [19].

Histology and Immunohistochemistry: Formalin-fixed, paraffin-embedded sections were prepared from liver tissue, then stained with hematoxylin and eosin or picrosirius red using standard techniques. For immunohistochemistry, antigen retrieval was performed by heating in 10 mM sodium citrate buffer, with a pH of 6.0, then specimens were incubated overnight with primary antibodies. The antibodies used were specific for smooth muscle α-actin (clone 1A4, #A5228, SMA, Sigma Chemical, St. Louis, MO, USA)) at 1:2000, or collagen type 1 (Abcam, Cambridge, MA, USA) #ab21286) at 1:250. Secondary horseradish peroxidase polymer-conjugated antibodies and detection were carried out using the EnVision kit (Agilent Dako, Santa Clara, CA, USA). Images were collected using a Nikon Eclipse 80i microscope and DS-Fi2 camera. For quantification of staining intensity, low-power images (2× objective) were subjected to histomorphometry using ImageJ software to apply color deconvolution and threshold functions, and data are expressed as the percent staining per total tissue area.

Cell culture: The LX-2 cell line, derived from human hepatic stellate cells, was generously provided by Dr. Scott Friedman (Icahn School of Medicine at Mount Sinai, New York, USA) [20]. The cells were maintained in a humidified 5% CO_2_ environment in Dulbecco’s modified Eagle’s medium supplemented with 2% fetal bovine serum. For experiments, cells were activated with 0.1 nM recombinant human TGF-β (R&D Systems, Minneapolis, MN, USA) overnight, then treated with 1 µM SR1664 or vehicle (0.05% dimethylformamide) for 16 h.

Gene expression assays: RNA was extracted from cell lysates using the PureLink Mini Kit (ThermoFisher, Waltham, MA, USA). The RNA was treated with DNase to remove the contaminating genomic DNA. The integrity and purity were determined by visualization on agarose gels. The RNA was quantified using the RiboGreen assay (ThermoFisher, Waltham, MA, USA), and 2 µg RNA was converted to cDNA using the High-Capacity cDNA Reverse Transcription Kit (Applied Biosystems, Waltham, MA, USA) in a total volume of 20 µL. For gene expression assays, 40 ng cDNA was used with TaqMan Universal Master Mix in a 20 µL reaction volume using a CFX Connect PCR system (Bio-Rad, Hercules, CA, USA). All primers and/or probe sets were designed to span introns. The human gene expression assays used (all from ThermoFisher, Waltham, MA, USA) included collagen type 1 (*COL1A2*), SMA (*ACTA2*), tissue inhibitor of matrix metalloproteinases-1 (*TIMP1*), tissue inhibitor of matrix metalloproteinases-2 (*TIMP2*), plasminogen activator inhibitor-1 (*SERPINE1*), transforming growth factor-β (*TGFB*), matrix metalloproteinase-1 (*MMP1*), matrix metalloproteinase-3 (*MMP3*), and glucuronidase-β (*GUSB*). The gene assay numbers and probe context sequences are provided in Table 1. The gene expression was normalized to that of *GUSB*, and the data expressed as expression relative to that of the control sample using the ΔΔCt method.

Western blotting: Whole-cell lysates were prepared using RIPA lysis buffer (20 mM Tris (pH 7.5) containing 150 mM NaCl, 1% Nonidet P40, 1% sodium deoxycholate, 0.1% sodium dodecyl sulfate (SDS), and Halt Phosphatase and Protease Inhibitor Cocktail (ThermoFisher Waltham, MA, USA). The total protein was quantified using the bicinchoninic acid (BCA) assay (ThermoFisher Waltham, MA, USA). A total of 25 µg protein was resolved on 10% polyacrylamide gels, then transferred to Immobilon-FL membranes (Millipore). Immunoblotting was performed using mouse anti-SMA antibody (#A5228, 1:2000, Sigma Chemical, St. Louis, MO, USA)) and rabbit anti-glyceraldehyde 3-phosphate dehydrogenase (GAPDH) antibody (#G9545, 1:10,000, Sigma Chemical, St. Louis, MO, USA), as well as secondary goat anti-mouse and goat anti-rabbit secondary antibodies labeled with IRDye 800 or IRDye 680, respectively (Li-Cor Biosciences, Lincoln, NE, USA). The blots were imaged using an Odyssey near-infrared scanner (Li-Cor Biosciences, Lincoln, NE, USA).

Cell proliferation assay: LX-2 cells were serum-deprived overnight in DMEM + 0.2% BSA (bovine serum albumin) in the presence of 1 µM SR1664 or vehicle. The cells were then stimulated with recombinant human platelet-derived growth factor BB (PDGF-BB, R&D Systems, Minneapolis, MN, USA) at 10 ng/mL, or vehicle (DMEM + 0.2% BSA) overnight. For the final 3 h of incubation, ^3^H-thymidine was added to the culture, then the DNA was precipitated using 10% trichloroacetic acid, solubilized in 0.1 M NaOH/0.1% SDS, mixed with Ultima Gold scintillation fluid, and counted in a liquid scintillation counter (PerkinElmer, Hopkinton, MA, USA).

Statistical analysis: All data are expressed as mean ± standard error. All graphs and statistical analyses were performed using Prism 8.0 software (GraphPad). Comparisons across multiple groups were conducted using one-way analysis of variance (ANOVA) with the Tukey post-test. Comparisons between two groups were made using the two-tailed Student *t*-test. In all cases, a value of *p* < 0.05 was considered significant.

## 3. Results

**Figure 1:**
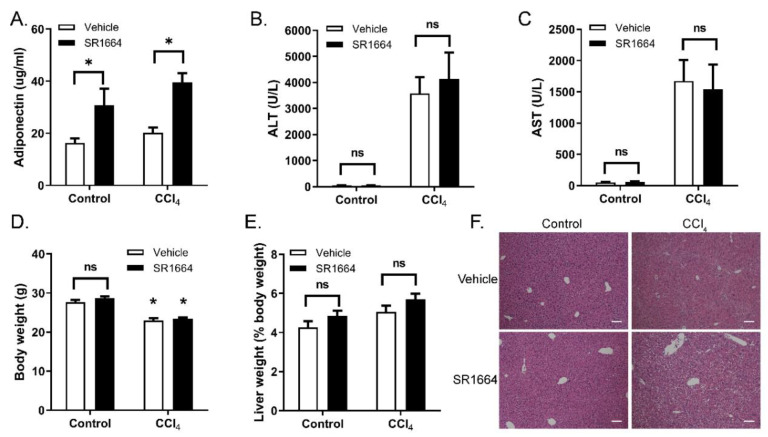
Serum determinations and histology from control or fibrotic mice treated with SR1664 or vehicle. (**A**) Serum adiponectin levels; (**B**,**C**) Serum ALT and AST levels; (**D**) Total body weight; (**E**) Liver weight expressed as percent total body weight; (**F**) Hematoxylin and eosin staining of liver sections. Bar = 200 µm; mean ± S.E.M; *n* = 6; * *p* < 0.05 by ANOVA; ns: not significant.

Hepatic fibrosis was induced in mice by treatment with CCl_4_ for 6 weeks, with or without SR1664 treatment for the final 2 weeks. To confirm the successful delivery and activity of SR1664, the adiponectin level was measured as a serum marker of PPARγ activation [21]. In both treatment groups, SR1664 resulted in significantly increased adiponectin levels, confirming that the oral administration of SR1664 resulted in biological activity in both control and CCl_4_-treated mice (Figure 1A). Treatment with CCl_4_ caused elevated serum ALT and AST levels compared to control mice (Figure 1B,C), which were unaffected by SR1664 treatment. The fibrotic mice also had reduced overall body weight compared to the control mice (Figure 1D), but there was no effect of SR1664. The liver weight as a percent of overall body weight was not significantly different in any group (Figure 1E). In histological samples stained with hematoxylin and eosin, the tissue architecture was disrupted in CCl_4_-treated mice, with bridging septae visible between the central veins (Figure 1F). In contrast, treatment with SR1664 resulted in decreased liver tissue damage. In non-fibrotic (control) mice, SR1664 had no significant effect on liver enzymes or histology. Therefore, treatment with SR1664 reduced the severity of histological liver tissue damage after fibrosis had been induced, but did not have an effect on serum markers of liver injury.

**Figure 2:**
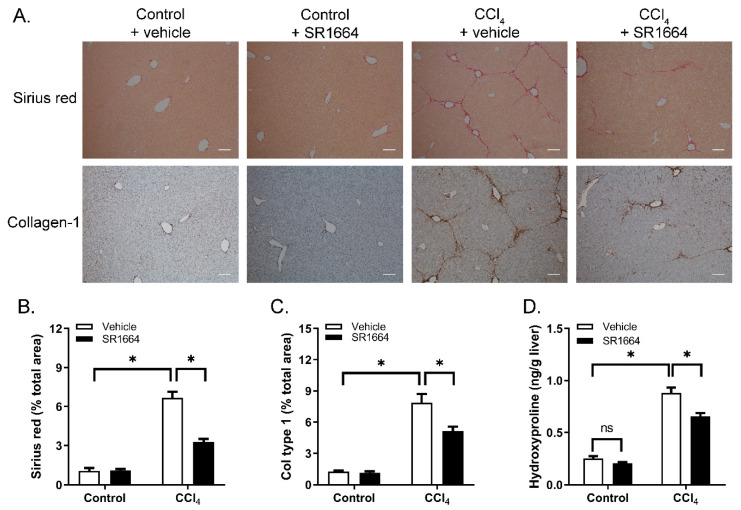
Collagen deposition in liver from control or fibrotic mice treated with SR1664 or vehicle. (**A**) Sirius red stain of total collagen (top panel) or immunohistochemistry for type 1 collagen (bottom panel); bar = 200 µm. (**B**) Quantification of Sirius red staining intensity.(**C**) Quantification of type 1 collagen staining. (**D**) Liver tissue hydroxyproline content. Mean ± S.E.M.; *n* = 6; * *p* < 0.05 by ANOVA; ns: not significant.

To determine if SR1664 treatment resulted in an altered liver collagen content, tissue sections were stained with Sirius red to visualize the fibrillar collagen (Figure 2A). In the control mice, staining was limited to the areas surrounding the major blood vessels. Treatment with CCl_4_ caused a large increase in the amount of collagen, particularly in the pericentral regions and extending into the parenchyma, with bridging between the central veins. Treatment with SR1664 caused a marked reduction in the Sirius-red-stained area. These findings were paralleled by using immunohistochemistry to detect type 1 collagen—a major constituent of excess collagen in hepatic fibrosis (Figure 2A). Quantification of both Sirius red and collagen immunohistochemistry revealed statistically significant reductions in response to SR1664 (Figure 2B,C). Finally, liver hydroxyproline content was measured as an estimate of total hepatic collagen. Treatment with CCl_4_ caused a large increase in hydroxyproline content, which was significantly reduced by SR1664 (Figure 2D). Taken together, the findings suggest that SR1664 was effective in reducing hepatic fibrosis.

**Figure 3:**
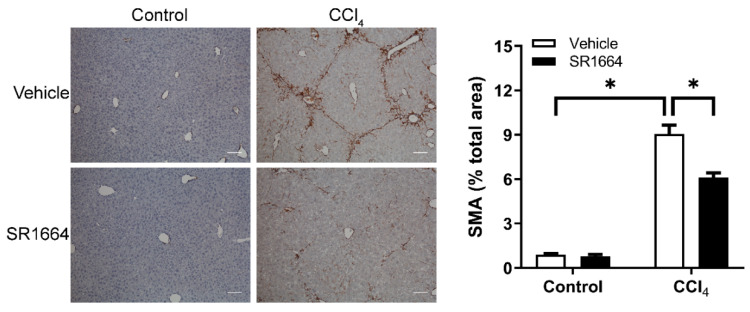
Smooth muscle α-actin (SMA) content in liver from control or fibrotic mice treated with SR1664 or vehicle. Immunohistochemistry for SMA (left panel); quantification of SMA staining intensity (right panel); bar = 200 µm; mean ± S.E.M.; *n* = 6; * *p* < 0.05 by ANOVA.

Liver injury results in the activation of HSCs, which produces most of the collagen in hepatic fibrosis. To determine if SR1664 affected HSCs’ activation, liver sections were subjected to immunohistochemistry to detect SMA, a marker of HSC activation. In the control mice, SMA was limited to the vascular smooth muscle cells (Figure 3). When treated with CCl_4_, the activated HSCs expressing SMA were detected in the pericentral zone, as well as in the areas of fibrotic bridging. This effect was significantly reduced in the livers of SR1664-treated mice. Therefore, SR1664 resulted in decreased activation of HSCs in hepatic fibrosis.

**Figure 4:**
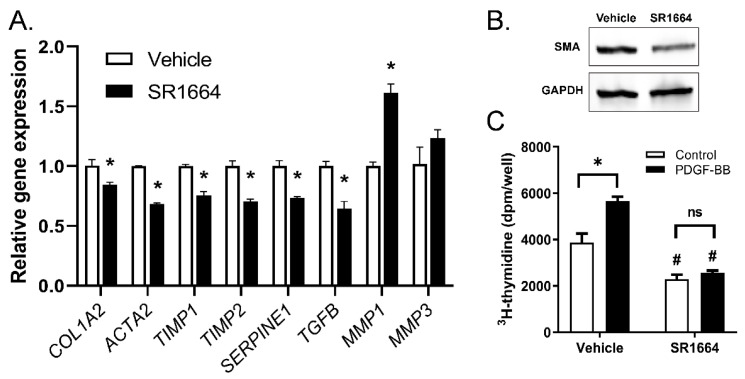
SR1664 had direct effects on hepatic stellate cells. (**A**) Gene expression in LX-2 cells treated with SR1664 or vehicle; (**B**) Western blot showing levels of SMA or GAPDH in LX-2 cells treated with vehicle or SR1664; (**C**) LX-2 cell proliferation in response to PDGF in the presence or absence of SR1664; mean ± S.E.M.; *n* = 3; * *p* < 0.05 compared with vehicle-treated cells; # *p* < 0.05 compared with vehicle or PDGF-treated cells. Significance determined by Student’s *t*-test (A) or ANOVA (C).

While SR1664 reduced fibrosis in vivo, it is not known whether these effects were a direct effect on the HSCs or an indirect effect through action on other cell targets. To determine if SR1664 has direct actions on HSCs, a human HSC-derived cell line, LX-2, was used. The cells were activated with TGF-β to promote a strong fibrotic phenotype, then the effect of SR1664 on the expression of fibrotic gene markers was determined (Figure 4A). Treatment with SR1664 significantly suppressed profibrotic gene markers of HSC activation, including type 1 collagen (*COL1A2*), SMA (*ACTA2*), PAI-1 (*SERPINE1*), and TGF-β (*TGFB*). In addition, SR1664 influenced genes to promote a collagen degrading phenotype, by suppressing the expression of the MMP inhibitors *TIMP1* and *TIMP2*, and increasing the expression of *MMP1*, a major fibrillary collagen-degrading protease. There was no significant effect on the expression of *MMP3*. Therefore, SR1664 changed the gene expression profile of LX-2 cells toward a less-activated phenotype. This finding was supported at the protein level, as the abundance of SMA was reduced as determined by Western blot (Figure 4B). Furthermore, SR1664 altered the cell proliferation of LX-2 cells, both at the basal level and in response to the potent hepatic stellate cell mitogen PDGF-BB (Figure 4C). Taken together, these findings suggest that the reduction in activated HSCs in vivo was due at least in part to the direct effects of SR1664 on HSCs.

## 4. Discussion

Previous studies showed that full PPARγ agonists such as troglitazone, pioglitazone, and rosiglitazone promoted antifibrotic phenotypes in cultured primary rat and human HSCs, including reduced cell proliferation, decreased expression of SMA and collagen, and increased MMP expression and activity [6,12,13,14]. The early in vivo studies in rat models of hepatic fibrosis also showed antifibrotic effects, but in all cases, these were prevention studies in which the treatment with the PPARγ agonists was begun prior to, or concomitantly with, the induction of fibrosis [6,7,8,9,10,11]. On the other hand, pioglitazone had no antifibrotic effect on the HSCs of mice and failed to prevent CCl_4_-induced hepatic fibrosis in mice [15]. In other studies performed with treatment models, in which the PPARγ agonists were administered after the establishment of significant fibrosis (a more clinically relevant model), the results were less favorable, as pioglitazone or rosiglitazone failed to reduce fibrosis in rodents subjected to CCl_4_ or cholestasis models of hepatic fibrosis [16,22]. Therefore, while drugs targeting PPARγ showed promise in the prevention of hepatic fibrosis, they appear to be ineffective at treating established fibrosis.

The reason for the failure of full PPARγ agonists such as the thiazolidinediones to reduce established fibrosis is not clear, but may be due to the strong transcriptional response triggered by these agents. In addition to the insulin-sensitizing effects, the activation of PPARγ triggers an adipogenic transcriptional program that can result in weight gain (largely due to increased adipose tissue mass), edema, and bone loss. For this reason, much attention has recently been paid to the development of selective or partial agonists of PPARγ, known collectively as selective PPARγ modulators (SPPARγMs) [23]. These agents act through alternative mechanisms to modulate PPARγ activity without activating the full transcriptional program seen with the full agonists. One class of these SPPARγMs (including SR1664) acts by binding to PPARγ to inhibit its inhibitory phosphorylation by CDK5, thereby unmasking a basal transcriptional activity that induces the genes associated with increased insulin sensitivity, but not the adipogenic gene program [17,18]. Therefore, these agents have the desirable antidiabetic effects without deleterious side effects such as edema, weight gain, and adiposity. However, the effect of these SPPARγMs on the antifibrotic effects triggered by PPARγ had not previously been explored.

In this study, the ability of SR1664 to influence hepatic fibrosis was determined in an established CCl_4_-induced model. Treatment with SR1664 did not affect the levels of ALT or AST, markers of hepatocyte death. This suggests that SR1664 does not protect hepatocytes from the acute toxic effects of CCl_4_ injection. However, SR1664 significantly reduced liver collagen content as assessed by total collagen staining, immunohistochemistry for collagen type 1, and liver hydroxyproline content, but had no effect on basal collagen content. This suggests that SR1664 can suppress the excess collagen triggered by chronic liver injury, but does not affect liver collagen levels under normal conditions.

Because PPARγ is widely expressed in many cell types, it is possible that the reduction in hepatic fibrosis was the result of both direct and indirect actions. Indeed, a serum marker for PPARγ activation by both full agonists and SPPARγMs is adiponectin, produced by the white adipose tissue. Adiponectin has antifibrotic effects on liver cells, including the inhibition of HSC activation [24]. Therefore, it is possible that the increased serum adiponectin in response to SR1664 was at least partially responsible for the reduced hepatic fibrosis in our study. However, it must be noted that adiponectin levels were also elevated in earlier studies showing a lack of effectiveness of rosiglitazone and pioglitazone on established fibrosis [16,22]. Therefore, increased adiponectin alone may not be responsible for the effects of SR1664. We therefore sought to determine if SR1664 had antifibrotic effects on cultured HSCs. We found suppression of profibrotic genes and elevation of *MMP1* after treatment with SR1664. Therefore, it is likely that direct action on the HSCs by SR1664 was at least partially responsible for the improvement in hepatic fibrosis in our study.

## 5. Conclusions

In summary, we have shown that a selective modulator of PPARγ was effective in reducing established hepatic fibrosis in a mouse model. It was also shown that SR1664 had direct effects on hepatic stellate cells, including the suppression of profibrotic genes, and induction of antifibrotic genes, providing a mechanism for at least part of this effect. Taken together, these studies support the continued development of SPPARγMs for the treatment of hepatic fibrosis and other diseases characterized by excess extracellular matrix deposition.

## Figures and Tables

**Table 1:** Gene expression assays.

Gene Symbol	Assay Number	Probe Context Sequence
*COL1A2*	Hs_01028970_m1	GCTGGCAGCCAGTTTGAATATAATG
*ACTA2*	Hs_00909449_m1	CTAAGACGGGAATCCTGTGAAGCAG
*TIMP1*	Hs_00171558_m1	TGAGGAATGCACAGTGTTTCCCTGT
*TIMP2*	Hs_00234278_m1	TCTCATTGCAGGAAAGGCCGAGGGG
*SERPINE1*	Hs_01126606_m1	TCATCCACAGCTGTCATAGTCTCAG
*TGFB*	Hs_99999918_m1	GACATCAACGGGTTCACTACCGGCC
*MMP1*	Hs_00233958_m1	TTTAAAGACAGATTCTACATGCGCA
*MMP3*	Hs_00968308_m1	AGAATTTGGGTTCTTTTATTTCTTT
*GUSB*	Hs_99999908_m1	ACTGAACAGTCACCGACGAGAGTGC
